# Reconstruction for Complex Oromandibular Facial Defects: The Fibula Free Flap and Pectoralis Major Flap Combination

**DOI:** 10.1155/2019/8451213

**Published:** 2019-03-26

**Authors:** Mohammed Qaisi, Ryan Dee, Issam Eid, James Murphy, Ignacio A. Velasco Martinez, Henry Fung

**Affiliations:** ^1^Oral-Head and Neck Oncology/Microvascular Surgery, Cook County Hospital, USA; ^2^Division of Oral and Maxillofacial Surgery, Cook County Hospital, USA; ^3^Division of Otolaryngology, Cook County Hospital, USA; ^4^Otolaryngology, St. John Clinic, Broken Arrow, OK, USA; ^5^Division of Plastic and Reconstructive Surgery, Rush University Medical Center, USA; ^6^Department of Oral and Maxillofacial Surgery and Pathology, University of Mississippi Medical Center, USA

## Abstract

**Background:**

Extensive through-and-through oromandibular defects after advanced oral carcinoma excision pose a reconstructive challenge for the head and neck surgeon. These complex oromandibular wounds often involve the mandible, oral and/or aerodigestive mucosa, and the external skin. As a result, these defects are often not amenable to reconstruction with a single flap due to the volume of soft tissue needed and the three-dimensional reconstructive requirement. The use of two free flaps has often been suggested to overcome this reconstructive challenge. A simpler and less technically demanding way to deal with this may involve the use of a free flap in combination with a pedicled regional flap. We present our experience of the use of a simultaneous microvascular fibula free flap (FFF) with a pectoralis major myocutaneous flap (PMMC) for addressing these defects.

**Methods:**

A retrospective chart review was performed of patients treated with a FFF and PMMC combination for the reconstruction of oromandibular defects at the University of Mississippi Medical Center (Jackson, MS) between October 2013 and February 2016. A minimum follow-up of 12 months was required. Data collected included the extent and location of tumor involvement, size of the postablative defect, tumor histology, clinical and pathological staging, length of follow-up, functional outcomes, and associated complications.

**Results:**

A total of three patients were identified to have been treated with the above technique. Defects repaired involved through-and-through mandibular defects with associated oral mucosa and external skin defects. In all cases, the FFF was used for restoring bony continuity with the skin paddle used to reconstruct the intraoral lining. The PMMC was used for reconstruction of the external skin defect and for providing soft tissue bulk. The average size of the fibula skin paddle used for intraoral reconstruction was 7.7 cm × 11.7 cm. The average size of the PMMC paddle was 7.3 × 9 cm. The mean follow-up was 21.7 months. Both the FFF and PMMC survived in all cases, although postoperative wound healing complications occurred in two of the three patients. There was one partial flap loss. Two patients regained good oral intake while one patient tolerated oral intake but was PEG tube-dependent.

**Conclusions:**

The combination of pectoralis major myocutaneous flap and a vascularized free fibular flap is a viable option for the reconstruction of complex through-and-through oromandibular defects. This technique may be useful when a single microvascular free flap is not sufficient for reconstruction of such defects.

## 1. Introduction

Through-and-through oromandibular defects may be defined as those involving the oral mucosa, mandible, and overlying skin. They are often a result of advanced pathology [1]. These types of defects present a reconstructive challenge for the head and neck surgeon [2]. While most head and neck ablative defects can be reconstructed with a single flap, limitations can arise when reconstructing large complex through-and-through oromandibular facial defects. In these extreme instances, a large skin paddle is needed intraorally. This makes closure of the external defect difficult, if not impossible, to reconstruct with the same flap. In these types of cases, some authors have advocated the use of two simultaneous free flaps, such as the fibula and radial forearm flap or fibula with anterolateral thigh flap combination [1–9]. This allows for reconstruction of all bony and soft tissue components and gives sufficient bulk for esthetics. The drawback of this technique is that two sets of microvascular anastomoses are necessary. Few authors in a previous series have reported on the use of the fibula free flap in combination with a regional flap such as the pectoralis major flap in the reconstruction of these defects [1, 10, 11]. In this manuscript, we share our experience of utilizing the fibula free flap (FFF) along with the pectoralis major myocutaneous flap (PMMC) for the reconstruction of these defects.

## 2. Methods

A retrospective chart review was performed of patients treated with a FFF and PMMC combination for the reconstruction of oromandibular defects at the University of Mississippi Medical Center between October 2013 and February 2016. Through-and-through oromandibular defects were defined as postablative defects that consisted of a mandibular discontinuity defect and both intraoral and external cutaneous soft tissue defects. Complete medical records and follow-up data for a minimum of 12 months were required. Data collected included a description of the extent and location of tumor involvement and treatment rendered, tumor histology, pathologic staging (AJCC 7th edition), length of follow-up, and functional outcomes. Data regarding the size of the FFF and PMMC skin paddles and the number of fibular segments used for reconstruction were also collected. Data regarding short-term and long-term complications were also recorded including the flap success rate, rate of flap take back, wound dehiscence, postoperative infections, and hardware failure.

In accordance with the policy of the institutional review board of the University of Mississippi Medical Center, the case series of three patients or less are exempt from institutional review board approval. Appropriate consent forms were obtained for patient photos used in this series. The authors declare that there is no conflict of interest regarding the publication of this paper.

## 3. Results

**Figure 1:**
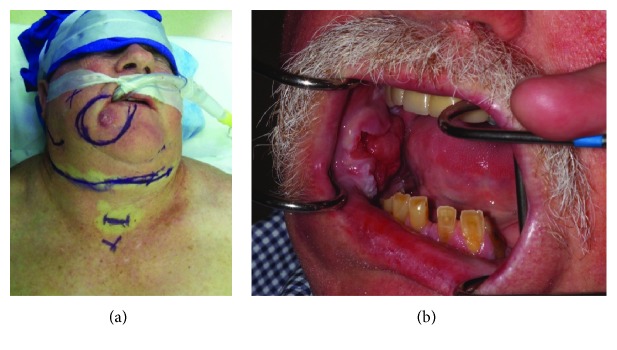
(a, b): preoperative extra and intraoral photos (patient 2 in Table 1). The carcinoma involved the right buccal mucosa with full thickness involvement of the right half of the lower lip and chin area. The lesion extends to involve the mandible and the maxillary gingiva.

**Figure 2 fig2:**
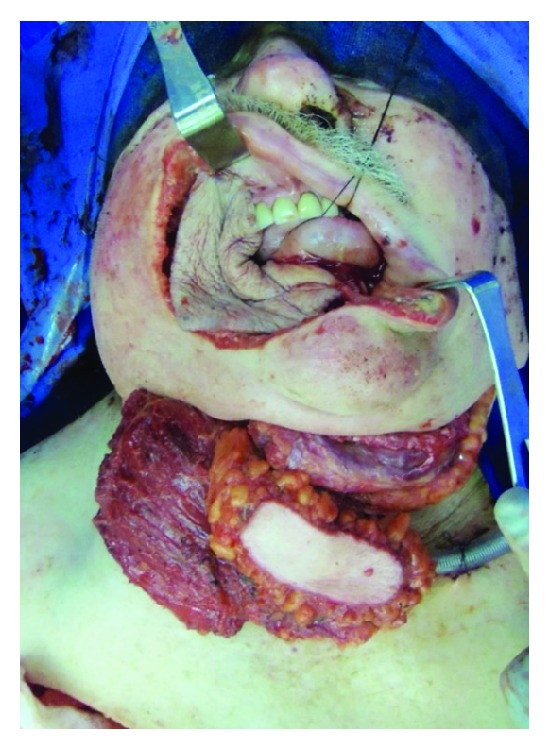
Intraoperative photo showing the skin paddle from the fibula flap being used to reline the intraoral cavity. The pectoralis flap has been tunneled into the neck and will be used for the reconstruction of the external skin defect.

**Figure 3:**
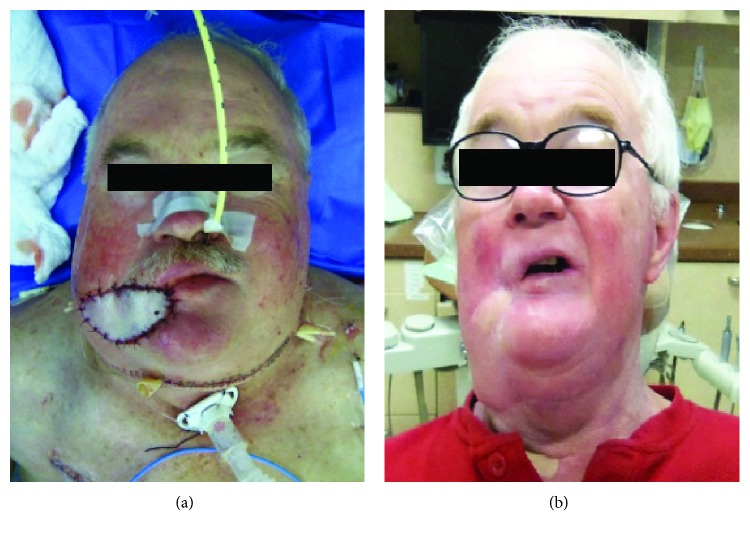
(a): the patient after the procedure. The lip defect was closed primarily to reestablish continuity of the vermillion. The fibula skin paddle was used for reconstructing the intraoral lining, and the pectoralis flap skin paddle was used for the external skin. (b): postoperative photo showing the patient 9 months after surgery.

A total of three patients with oromandibular defects were treated with a FFF and PMMC during this time frame. Defects repaired involved through-and-through mandibular defects with associated oral mucosa and external skin defects. Patient 1 had a pT2 N0 M0 G3 mandibular high-grade osteosarcoma with involvement of the overlying skin. Patient 2 (index case) had a right pT4a N0 M0 buccal squamous cell carcinoma with invasion of the mandible and maxilla and induration and involvement of the overlying skin in the chin and cheek area (Figures 1 [Fig fig2]–3, Video 1). Patient 3 had similar findings with a pT4a N2b squamous cell carcinoma of the left mandible and floor of the mouth. Although bony defects extended well beyond the midline in 2 of 3 patients, the external cutaneous defects were localized to one side. A summary and description of the defects are found in Table 1.

In all three cases, the surgical technique involved the use of the FFF for restoring bony continuity with the skin paddle used to reconstruct the intraoral lining. The PMMC was used for reconstruction of the external skin defect and for providing soft tissue bulk (Figures 1 [Fig fig2]–3). The vessels in all three cases were anastomosed to vessels in the contralateral neck because mandibular reconstruction extended to or beyond the midline and in order to avoid compression by the overlying PMMC flap. The average size of the fibula skin paddle used for intraoral reconstruction was 7.7 cm × 11.7 cm. The average size of the PMMC paddle used for the external defects was 7.3 × 9 cm. The number of fibular segments utilized in the mandibular reconstruction in patients 1–3 was 3, 1, and 2, respectively. The mean follow-up was 21.7 months. All patients underwent adjuvant therapy. Patients 1 and 2 underwent radiotherapy, and patient 3 underwent concurrent chemoradiation. All patients were alive and free of disease at their last follow-up.

Both the FFF and PMMC survived in all cases. There were no take backs to the operating room for complications related to venous congestion or flap thrombosis. Early postoperative complications developed in patients 2 and 3 with wound breakdown occurring at the distal aspect of the PMMC skin paddle. This was managed with conservative therapy, wound care, and resuturing of the area when necessary. Patient 3 developed exposure of the middle portion of the posterior fibular segment intraorally after the completion of adjuvant chemoradiotherapy. This may represent a partial flap failure versus osteoradionecrosis of the fibula. The posterior fibular segment was still exposed at 17 months, but the patient did not wish to undergo any further surgery and was keeping the area clean with daily irrigations.

A late complication occurred in patient 1 which involved a <1 cm exposure of the mandibular hardware at 14 months of follow-up. This was corrected with partial removal of the hardware via a transoral approach, and the patient had uneventful healing. There were no additional complications until the last follow-up at 27 months.

All patients had intelligible speech after surgery. In terms of swallowing, patients 1 and 2 regained good oral intake while patient 3 tolerated liquids but was PEG tube-dependent for meeting nutritional needs.

## 4. Discussion

While most head and neck defects can be reconstructed with a single free flap, large through-and-through oromandibular facial defects can present a reconstructive challenge. These defects often involve the mandible, oral mucosa, and external cutaneous skin, resulting in significant loss of the mandibular bone and soft tissue bulk. Due to the extensive soft tissue requirement and three-dimensional complexity of these wounds, it is often difficult to select a single donor site that can restore such defects. The combination of pectoralis major myocutaneous flap and a vascularized free fibular flap offers a viable and less technically demanding alternative to the use of 2 microvascular flaps. Although previously documented, a limited number of series have looked at this treatment modality [2, 10–13]. Our goal was to share our experience with this previously described technique.

In the series by Chen et al., a total of 14 patients were treated using this technique. Similar to our technique, they used the fibula for bony reconstruction and the skin paddle for the mucosal defect. The pectoralis major flap was used for reconstruction of the external cutaneous defect. There was 1 flap failure in the series and 2 salvaged reexplorations (15.4%) [10]. They questioned whether compression from the pectoralis flap could have led to venous congestion in these cases. As previously discussed, in our series, the vascular anastomosis was performed in the contralateral neck to avoid compression from the overlying PMMC flap. No reexplorations were necessary due to venous congestion in our limited series. The use of contralateral vessels may be something to consider when using this flap combination. At a minimum, special attention needs to be paid to vessel geometry and avoiding compression from the overlying PMMC flap.

One advantage of this technique is the ease of harvest of the widely accepted PMMC flap even for the novice surgeon [14]. The PMMC flap is reliable with less than 10% of the major complication rate requiring reoperation. Its main limitation has to do with the arc of rotation [15]. A previous series suggested that this technique be reserved for through-and-through defects located more laterally due to being limited by the arc of rotation of the PMMC [1, 5]. We are in agreement with this. While in our series, we were able to reconstruct mandibular and intraoral defects that extended well beyond the midline; the external skin defects were primarily restricted to the ipsilateral side.

The PMMC can be associated with a tenuous blood supply of the distal skin paddle which may result in partial wound breakdown, dehiscence, fistulation, and infection which may prolong the hospital stay [14, 16]. In our series, patients 2 and 3 both developed tissue dehiscence at the distal end of PMMC flap skin paddle which resolved with local wound care and primary closure at 4 weeks as discussed previously. The patients were able to start adjuvant therapy on time.

Other complications that were encountered in our patients included postoperative dysphagia. Patient 3 developed postoperative dysphagia, and although he was able to tolerate liquids by mouth, he was dependent on tube feeds for nutritional support. The etiology for this is likely multifactorial. His surgical resection included the floor of the mouth, part of the tongue, and a large portion of the mandible. This combined with the postoperative concurrent chemoradiation and bulkiness of the reconstruction may all have contributed to the dysphagia. Other long-term complications included hardware exposure through native radiated facial skin at 14 months in patient 1. This was rectified by segmental removal of the hardware via a transoral approach, and this healed uneventfully. Finally, there was the partial exposure of the posterior fibular segment in patient 3 after the completion of concurrent chemoradiation. This may represent partial flap loss versus osteoradionecrosis of the fibula. The patient was not interested in having this bony fibular segment removed and continued to have partial exposure of this segment, which was managed with daily irrigations.

We believe that the complication profile for patients in this cohort is no different from other patients that we treat with advanced stage head and neck cancer. These patients often undergo extensive surgery and require multimodality treatment, and a certain degree of complications is to be expected [17]. In a review of similar patients at Chang Gung Memorial Hospital, they presented a series of patients with T3/T4 squamous cell carcinoma resections that resulted in large through-and-through defects. The authors report complications including partial skin loss of the distal pectoralis major flap and an exposed plate following radiotherapy. They also reported 1 total free flap failure and 2 salvaged reexplorations among 13 surviving free flaps in their series, which were attributed to venous obstruction. The overall complication rate for the pectoralis major flap was 21. 4% and that for the fibular osteoseptocutaneous flap was 14.3%. They concluded that the FFF/PMMC flap was successful and a technically less demanding alternative to the double free flap procedure in the reconstruction of extensive lateral mandibular defects [10]. In another series, the overall flap success rate was 94%, and the overall complication rate was 50%. Staged and ancillary reconstruction procedures were required in 63% of patients [11].

## 5. Conclusion

Large through-and-through oromandibular defects present a difficult reconstructive challenge. The combination of pectoralis major myocutaneous flap and a vascularized free fibular flap provides a viable option for the reconstruction of these defects.

## Figures and Tables

**Table 1:** Summary of patient description, tumor characteristics, treatment rendered, and outcomes.

	Age	Pathology	Stage	Months of follow up	Site involved-resultant defect	Reconstruction	# of fibular segments	Size of fibula skin paddle	Size of pectoralis major myocutaneous paddle	Functional outcome	Complications
Patient 1	20 year old African American Male	High Grade Osteosarcoma	T2N0M0G3	29 months	10 × 9 cm lesion right mandible extending form right subcondylar area to left premolar region, involved floor og mouth, buccal mucosa and right cheek and neck skin	Free fibular flap with skin paddle intraorally and pectoral major myocutaneous skin paddle for external defect	3	7 × 12 cm	11 × 9 cm	Excellent. Good oral intake. Mouth opening 40 mm	Hardware exposure at 14 months. Treated with hardware removal.
Patient 2	65 year old male	Squamous cell carcinoma	T4aN0	19 months	Right buccal mucosa lesion extending to maxillary gingiva and mandible, with full thickness involvement of the right half of lower lip and cheek/chin area	Free fibular flap with skin paddle intraorally and pectoral major myocutaneous skin paddle for external defect	1	7 × 9 cm	6 × 5 cm	Good oral intake. Microstomia due to lip resection and post operative adjuvant therapy	Delayed wound healing at distal end of pectoralis major myocutaneous skin paddle. Managed succesfully with wound care. At 16 months underwent commissurotomy to try to improve microstomia, however developed wound breakdown. Moved to another city at 19 months
Patient 3	53 year old American male	Squamous cell carcinoma	T4aN2c	17 months	Left mandible extending from right premolar region, included floor of mouth, buccal mucosa, lower lip, chin and neck skin	Free fibular with skin paddle intraorally and pectoral major myocutaneous skin paddle for external defect	2	9 × 4 cm	10 × 8 cm	Percutaneous tube dependent, with some oral intake. Microstomia due to lip resection and adjuvant therapy	Delayed healing at distal end of pectoralismajor myocutaneous skin paddle. Managed successfully with wound care. Following chemo and radiation developed partial exposure of the left posterior fibular segment. Patient elected to observe area.

## Supplementary Materials

Supplementary Materials Video 1: video with commentary describing this surgical technique used in the reconstruction of complex oromandibular defects.
